# Social correlates of term small for gestational age babies in a Russian Arctic setting

**DOI:** 10.3402/ijch.v75.32883

**Published:** 2016-11-29

**Authors:** Anna A. Usynina, Andrej M. Grjibovski, Jon Øyvind Odland, Alexandra Krettek

**Affiliations:** 1 Department of Community Medicine, Faculty of Health Sciences, UiT The Arctic University of Norway, Tromsø, Norway; 2 International School of Public Health, Northern State Medical University, Arkhangelsk, Russia; 3 Department of International Public Health, Norwegian Institute of Public Health, Oslo, Norway; 4 Department of Preventive Medicine, International Kazakh-Turkish University, Turkestan, Kazakhstan; 5 Department of Public Health, Hygiene and Bioethics, Institute of Medicine, North-Eastern Federal University, Yakutsk, Russia; 6 Department of Public Health, Faculty of Health Sciences, University of Pretoria, Pretoria, South Africa; 7 Department of Biomedicine and Public Health, School of Health and Education, University of Skövde, Skövde, Sweden; 8 Department of Internal Medicine and Clinical Nutrition, Institute of Medicine, Sahlgrenska Academy at University of Gothenburg, Gothenburg, Sweden

**Keywords:** birth registry, light for date, SGA, small for date, Russia

## Abstract

**Background:**

Small for gestational age (SGA) births have been associated with both short- and long-term adverse health outcomes. Although social risk factors for SGA births have been studied earlier, such data are limited from Northern Russia.

**Objective:**

We assessed maternal social risk factors for term SGA births based on data from the population-based Murmansk County Birth Registry (MCBR).

**Design:**

Data on term live-born singleton infants born between 2006 and 2011 in Murmansk County were obtained from the MCBR. We applied the 10th percentile for only birth weight (SGA_W_) or for both birth weight and birth length (SGA_WL_). Binary logistic regression was used to estimate the effect of independent variables on SGA males and females with adjustment for known risk factors and potential confounders. Both crude and adjusted odds ratios with 95% confidence intervals for the studied risk factors were calculated.

**Results:**

The proportions of term SGA_W_ and SGA_WL_ births were 9.7 and 4.1%, respectively. After adjustment for potential confounders, the risk of term SGA births among less educated, unemployed, unmarried, smoking and underweight women was higher compared with women from the reference groups. Evidence of alcohol abuse was also associated with birth of SGA_WL_ and SGA_W_ boys. Maternal overweight and obesity decreased the risk of SGA.

**Conclusions:**

Maternal low education, unemployment, unmarried status, smoking, evidence of alcohol abuse and underweight increased the risk of term SGA births in a Russian Arctic setting. This emphasizes the importance of both social and lifestyle factors for pregnancy outcomes. Public health efforts to reduce smoking, alcohol consumption and underweight of pregnant women may therefore promote a decrease in the prevalence of SGA births.

Small for gestational age (SGA) birth is an unfavourable pregnancy outcome and contributes to both short- and long-term adverse health effects for children born SGA. To date, many risk factors of SGA births have been reported.

## Current definitions of SGA birth

The International Classification of Diseases, 10th Revision (ICD-10) defines SGA birth as a birth with infant birth weight (BW) and birth length (BL) below the 10th percentile (P10) for gestational age (GA) (ICD-10 code P05.1) (1). In some publications, SGA babies have also been defined as those born with either low BW (SGA_W_) or length (SGA_L_) or both low BW and BL (SGA_WL_) for GA (2, 3). This classification helps to better understand the aetiology and mechanisms, as well as health effects of being born SGA. An additional definition of SGA as the sex- and GA-specific reference mean for BW and/or BL below two standard deviations (SDs) was recommended to identify children for future growth-promoting interventions (3).

## Short- and long-term outcomes of SGA births

Compared with appropriate weight for GA births, infants born SGA have higher risk of perinatal, early neonatal (4), as well as infant and child mortality (3). In addition, SGA infants exhibit an increased risk of developing hyperactivity disorders (5), neurodevelopmental delay and persistent short stature later in life (6). Term SGA infants have lower scores on neurodevelopmental outcomes (7, 8) and problems in scholastic/vocational attainments (9) compared with term non-SGA infants. Compared with individuals born with appropriate weight for GA, adults born SGA are at increased risk of cardiovascular (10) and metabolic disorders (11), behavioural problems, lower intelligence and social competence, and poor academic performance (12).

## Social risk factors influencing SGA

Maternal cigarette smoking (13, 14), obesity, advanced age (≥35 years) and null parity (13) are established risk factors for SGA. Unmarried status, maternal young age (<20 years) and low education contribute to term SGA birth (4). Maternal low body mass index (BMI) (15, 16), poor nutrition in pregnancy (17), urban residence (18) and alcohol consumption (16, 19) also increase the risk of SGA. An association between both short and long inter-pregnancy intervals and SGA births has been reported (20, 21). However, an association between specific parental occupation and risk of SGA birth remains unclear. Maternal and/or paternal unemployment (22) and high unemployment rate in neighbourhoods (23) associate with higher risk of SGA. Paternal occupation likely does not impact on SGA birth. In contrast, mothers working as electrical or textile workers (24, 25) as well as beverage manufacture workers (25) are at higher risk of SGA birth. However, being employed as a nurse associates with lower risk of SGA (26). Living in a low-income neighbourhood also associates with increased risk of SGA birth (27).

## Studies of SGA birth in Russia

Data based on the Kola Birth Registry, implemented in the city of Monchegorsk in Northwest Russia, demonstrate an SGA_W_ prevalence of 9.2% during 1973–2003. The proportion of SGA infants is highest among unemployed women/homemakers (28). In Tula County in Central Russia, secondary specialized and higher maternal education (either complete or not) associates with a higher child mean BW. In addition, married mothers have children with higher BW compared with single mothers, and higher infant BW is observed in ethnic Russians than in non-Russians (29). Such data are in line with results of the Severodvinsk study in Northern Russia regarding influence of mothers’ education level on BW. Here, heavier infants were born to more educated women (30). Smokers and alcohol abusing mothers, as well as those perceiving stress or living in poor conditions, are at increased risk of delivering lighter babies (31).

To date, no reference measures of BW and BL for different GAs as well as investigations of the socio-demographic risk factors for SGA births based on birth registry data are available in a Russian Arctic setting. The purpose of our study was therefore to create cut-off values for BW and BL for term SGA births and to assess maternal social risk factors for SGA births using data from a population-based registry.

## Materials and methods

### Study design and data collection

We conducted a registry-based cohort study with data from the Murmansk County Birth Registry (MCBR). Murmansk County is situated in Northwest Russia. Data collection in the MCBR began on 1 January 2006 and continued until 31 December 2011. The Registry contains socio-demographic information, data about the index pregnancy and mother's pre-pregnancy health, delivery and infant's health. A set of previously published studies describes MCBR in detail (32–34).

**Fig. 1:**
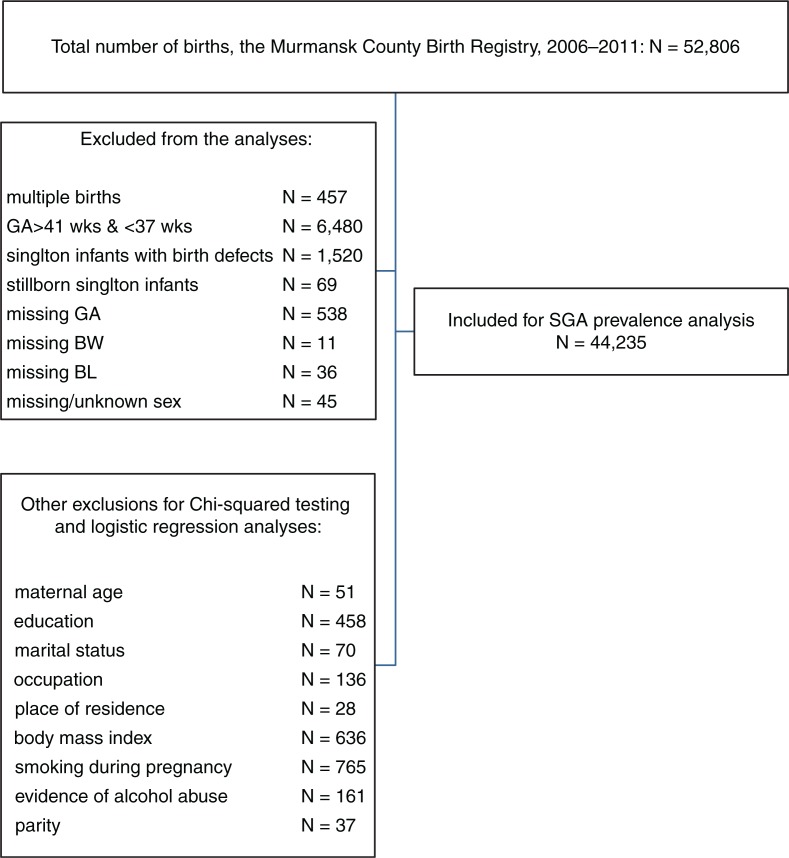
Selection procedure of the study population. The figure shows the number of births recorded in the Murmansk County Birth Registry and those selected for analyses. BL, birth length; BW, birth weight; GA, gestational age; SGA, small for gestational age; wks, weeks.

The study population included 52,806 births. We excluded multiple births, stillborn infants and those born with a birth defect reported at birth as well as records with missing data on BW, BL, GA and missing or unknown infant sex (altogether 8,571 births). GA was determined on the basis of first ultrasound (US) in pregnancy. We used last menstrual period (LMP) to estimate GA in 4,001 births with missing US data. Births before 37 and after 41 weeks of gestation were also excluded from the analyses. A total of 44,235 births were included in SGA_W_ and SGA_WL_ births’ prevalence analyses and the analyses of percentiles of BW and BL for each gestational week between 37 and 41. We excluded 1,996 records with missing data on studied independent variables or potential confounders to perform further logistic regression analyses. Our final study sample included 42,239 births. The algorithm of sampling is presented in Fig. 1.

## Data analysis

### Outcome variable

We used SGA as a dichotomous dependent variable. The 10th percentile for BW or both BW and BL for each gestational week between 37 and 41 was applied to classify, respectively, SGA_W_ and SGA_WL_ births.

### Exposure variables

In our study, marital status, mother's age, education, BMI, smoking, evidence of alcohol abuse during pregnancy, place of residence and occupation were used as independent variables. Maternal age was divided into three groups: <18, 18–34 and ≥35 years. We selected maternal age of 18–34 years as reference group. Maternal education was categorized as none or primary (Grades 1–9), secondary (Grades 10 and 11), vocational school and higher with the last one as the reference category. By marital status, all mothers were divided into two groups: married and unmarried. Unmarried included single, cohabiting, divorced or widowed. Maternal occupation status was divided into employed/student (including pupils) and unemployed. Mothers’ BMI was categorized as underweight (BMI<18.5 kg/m^2^), normal weight (BMI=18.5–24.9 kg/m^2^), and overweight and obese (BMI≥25.0 kg/m^2^). Mothers were divided into non-smokers and smokers according to their smoking status during pregnancy. Evidence of mother's alcohol abuse during pregnancy was recorded as “no” or “yes.” Place of mother's residence was dichotomized into rural and urban, with rural residence serving as the reference group.

### Statistical analyses

We used chi-squared tests to study differences in distribution of selected risk factors in SGA and non-SGA birth groups for both definitions of SGA. When comparing SGA_W_ and non-SGA_W_ groups, all variables except place of residence had significantly different distributions and were included in multivariable regression analysis.

Binary logistic regression was used to estimate the effect of studied variables on SGA_W_ and SGA_WL_. Initially, we applied two regression models to separately estimate the effect of studied factors on SGA_W_ and SGA_WL_ births and those were additionally adjusted for parity. We examined the regression models with both outcomes, SGA_W_ and SGA_WL_ births, for multicollinearity, but found no effect of such collinearity. We also checked for interactions between all variables in the regression models with both SGA_W_ and SGA_WL_ births as outcomes. The interactions were non-significant, with the exception of an interaction between smoking and education in a model with SGA_W_ as dependent variable as well as between maternal age and employment in the model with SGA_WL_ as an outcome. Therefore, we used categories smoker or non-smoker for each category of education in our final regression models for SGA_W_ birth for both sexes. In regression analyses for risk factors of SGA_WL_ births for male and female infants, we used categories employed or unemployed women in each of the three maternal age groups. We calculated both crude and adjusted odds ratios (ORs) with 95% confidence intervals for the studied risk factors separately for male and female infants. Analysis was done with IBM SPSS Statistics for Macintosh, Version 23.0 (IBM Corporation, Armonk, NY).

## Ethics and consent

The MCBR registration forms do not contain personal identifiers, which means that the health information is confidential and therefore no personal consent was needed. The study received ethical approvals from the Ethical Committee of the Northern State Medical University (Arkhangelsk, Russia) (Protocol 04/5-13) and the Regional Committee for Medical and Health Research Ethics in Northern Norway (2013/2300 REK nord).

## Results

Data on sex-specific infant BW and BL percentiles by GAs are presented in Table I. Boys were heavier than girls at each studied GA. The 10th percentile for BL in boys was also higher between 38 and 39 weeks of GA. In our study, 4.1 and 9.7% of all births were classified as SGA_WL_ and SGA_W_, respectively (Table II). We found that the proportions of SGA_WL_ girls were higher compared with SGA boys at GA of 37, 40 and 41 weeks.

**Table I:** Tenth percentile values for BW and BL for singleton term births both sexes in Murmansk County, Russia during 2006–2011

	Number of births		BW 10th percentile (g)		BL 10th percentile (cm)	
			
Gestational age (weeks)	Male	Female	Male	Female	Male	Female
37	1,646	1,428	2,617	2,529	48	48
38	4,011	3,679	2,810	2,680	49	48
39	7,304	6,763	2,970	2,850	50	49
40	7,072	6,762	3,050	2,950	50	50
41	2,722	2,848	3,103	2,960	50	50

BL, birth length; BW, birth weight; cm, centimetre; g, gram.

**Table II:** Number of SGA_WL_ and SGA_W_ births by infant sex and gestational age in Murmansk County, Russia during 2006–2011

	Number of SGA_WL_ births, n (%)		Total number of SGA_WL_ births, n (%)	Number of SGA_W_ births, n (%)		Total number of SGA_W_ births, n (%)
		
Gestational age (weeks)	Male	Female		Male	Female	
37	66 (4.0)	85 (6.0)	151 (4.9)	164 (10.0)	142 (9.9)	306 (10.0)
38	172 (4.3)	112 (3.0)	284 (3.7)	400 (10.0)	367 (10.0)	767 (10.0)
39	355 (4.9)	252 (3.7)	607 (4.3)	712 (9.7)	644 (9.5)	1,356 (9.6)
40	229 (3.2)	349 (5.2)	578 (4.2)	663 (9.4)	646 (9.6)	1,309 (9.5)
41	76 (2.8)	139 (4.9)	215 (3.9)	272 (10.0)	275 (9.7)	547 (9.8)
Total number	937 (4.0)	898 (4.3)	1,835 (4.1)	2,211 (9.7)	2,074 (9.7)	4,285 (9.7)

n, number of cases; SGA_WL,_ small for gestational age defined as both birth weight (BW) and length <10th percentile; SGA_W_, small for gestational age defined as BW <10th percentile.

Compared with non-SGA births, both the SGA_WL_ and SGA_W_ birth groups had higher proportion of young, single mothers, women with low educational level, unemployed and underweight women (Table III). Overweight and obese mothers were less likely to give birth to both SGA_WL_ and SGA_W_ babies. Rural residence was higher in SGA_WL_ compared with non-SGA_WL_; however, the proportion of mothers living in rural and urban areas in SGA_W_ and non-SGA_W_ groups was not statistically different. The proportion of smokers was twice as high in the SGA birth groups compared to non-SGA births. Evidence of alcohol abuse was also higher in SGA when compared with non-SGA births.

**Table III:** Proportions of SGA_WL_ and SGA_W_ births by maternal social characteristics and potential confounders in Murmansk County, Russia during 2006–2011

	Births, n=44,235			Births, n=44,235		
				
Characteristic	Non-SGA_WL_ births, n (%)	SGA_WL_ births, n (%)	p*	Non-SGA_W_ births, n (%)	SGA_W_ births, n (%)	p**
Age (years)						
<18	558 (1.3)	41 (2.2)	<0.001	502 (1.3)	97 (2.3)	<0.001
18–34	38,095 (90.0)	1,661 (90.6)		35,882 (89.9)	3,874 (90.5)	
35+	3,698 (8.7)	131 (7.1)		3,519 (8.8)	310 (7.2)	
Education						
None or primary	1,252 (3.0)	140 (7.7)	<0.001	1,126 (2.8)	266 (6.3)	<0.001
Secondary	13,848 (30.6)	769 (42.4)		11,928 (30.2)	1,689 (39.9)	
Vocational school	13,383 (31.9)	542 (29.9)		12,657 (32.0)	1,268 (29.9)	
Higher	14,481 (34.5)	362 (20.0)		13,828 (35.0)	1,015 (23.9)	
Occupation						
Unemployed	12,224 (28.9)	798 (43.7)	<0.001	11,333 (28.5)	1,689 (39.6)	<0.001
Employed/students	30,049 (71.1)	1,028 (56.3)		28,499 (71.5)	2,578 (60.4)	
Marital status						
Unmarried	10,644 (25.1)	757 (41.4)	<0.001	9,832 (24.6)	1,569 (36.7)	<0.001
Married	31,693 (74.9)	1,071 (58.6)		30,059 (75.4)	2,705 (63.3)	
Place of residence						
Urban area	32,471 (76.6)	1,347 (73.4)	0.002	30,568 (76.6)	3,250 (75.9)	0.316
Rural area	9,902 (23.4)	487 (26.6)		9,356 (23.4)	1,033 (24.1)	
Nutritional status						
Underweight	2,551 (6.1)	182 (10.2)	<0.001	2,276 (5.8)	457 (10.9)	<0.001
Normal weight	27,565 (65.9)	1,242 (69.7)		25,911 (65.7)	2,896 (69.3)	
Overweight and obese	11,700 (28.0)	359 (20.1)		11,231 (28.5)	828 (19.8)	
Smoking during pregnancy						
No	34,552 (82.9)	1,139 (63.6)	<0.001	32,767 (83.4)	2,924 (69.6)	<0.001
Yes	7,126 (17.1)	653 (36.4)		6,501 (16.6)	1,278 (30.4)	
Evidence of alcohol abuse						
No	42,147 (99.8)	1,802 (98.5)	<0.001	39,734 (99.8)	4,215 (98.9)	<0.001
Yes	98 (0.2)	27 (1.5)		76 (0.2)	49 (1.1)	

* p-values indicate that differences in proportion exist between SGA_WL_ and non-SGA_WL_ births for the indicated characteristics;

** p-values indicate that differences in proportion exist between SGA_W_ and non-SGA_W_ births for the indicated characteristics. N, number of cases; SGA_WL_, small for gestational age defined as both birth weight (BW) and length <10th percentile; SGA_W_, small for gestational age defined as BW <10th percentile.

In crude analyses, we found significant association between all studied characteristics (marital status, maternal age, education, nutritional status, smoking, evidence of alcohol abuse and occupation) with SGA births (Tables IV and V). Urban residence was associated with decreased risk of SGA_WL_ births. The risk of both SGA_WL_ and SGA_W_ births in overweight and obese women was lower compared with normal weight mothers. The results were similar for male and female SGA infants. Older (≥35 years) women had lower risk to deliver SGA_W_ boys and girls compared with 18–34 years old mothers; hence, the association between advanced maternal age and decreased risk of SGA_W_ males has not reached statistical significance (Table V).

**Table IV:** Results of multivariable regression analyses for risk factors of SGA_WL_ births in Murmansk County, Russia during 2006–2011

	SGA_WL_, males, n=840			SGA_WL_, females, n=872		
		
Characteristic	Crude OR	Adjusted OR^a^ (95% CI)	Adjusted OR^b^ (95% CI)	Crude OR	Adjusted OR^a^ (95% CI)	Adjusted OR^b^ (95% CI)
Age (years) in unemployed/employed mothers						
<18, unemployed	1.91 (0.97–3.76)	0.81 (0.40–1.65)	0.76 (0.38–1.55)	3.23 (1.99–5.24)	1.38 (0.82–2.33)	1.36 (0.81–2.30)
<18, employed/students	1.65 (0.72–3.77)	0.76 (0.33–1.78)	0.71 (0.31–1.67)	1.22 (0.49–2.99)	0.47 (0.19–1.17)	0.46 (0.18–1.15)
18–34, unemployed	1.89 (1.63–2.19)	1.42 (1.21–1.66)	1.41 (1.20–1.65)	1.67 (1.44–1.93)	1.16 (0.99–1.36)	1.16 (0.99–1.36)
18–34, employed/students	1.00	1.00	1.00	1.00	1.00	1.00
35+, unemployed	2.84 (1.82–4.44)	2.58 (1.63–4.09)	2.79 (1.75–4.44)	1.28 (0.73–2.25)	1.21 (0.68–2.14)	1.23 (0.69–2.19)
35+, employed/ students	0.83 (0.60–1.15)	0.91 (0.65–1.26)	0.98 (0.70–1.36)	0.75 (0.55–1.04)	0.92 (0.66–1.27)	0.94 (0.67–1.30)
Education						
None or primary	4.12 (3.07–5.72)	2.24 (1.58–3.17)	2.28 (1.61–3.23)	4.37 (3.26–5.86)	2.30 (1.65–3.21)	2.31 (1.66–3.23)
Secondary	2.30 (1.91–2.77)	1.48 (1.20–1.81)	1.49 (1.22–1.83)	2.47 (2.06–2.97)	1.72 (1.41–2.11)	1.73 (1.41–2.11)
Vocational school	1.63 (1.34–1.98)	1.31 (1.07–1.61)	1.33 (1.08–1.62)	1.61 (1.32–1.95)	1.36 (1.11–1.66)	1.36 (1.16–1.66)
Higher	1.00	1.00	1.00	1.00	1.00	1.00
Marital status						
Unmarried	2.11 (1.83–2.43)	1.63 (1.41–1.90)	1.60 (1.38–1.86)	2.07 (1.80–2.38)	1.52 (1.31–1.76)	1.51 (1.31–1.76)
Married	1.00	1.00	1.00	1.00	1.00	1.00
Place of residence						
Urban area	0.84 (0.72–0.98)	0.91 (0.78–1.07)	0.90 (0.77–1.06)	0.83 (0.71–0.96)	0.92 (0.79–1.08)	0.92 (0.79–1.08)
Rural area	1.00	1.00	1.00	1.00	1.00	1.00
Nutritional status						
Underweight	1.53 (1.20–1.95)	1.43 (1.12–1.82)	1.41 (1.10–1.80)	1.62 (1.29–2.02)	1.47 (1.17–1.85)	1.47 (1.17–1.85)
Normal weight	1.00	1.00	1.00	1.00	1.00	1.00
Overweight and obese	0.77 (0.65–0.91)	0.77 (0.65–0.91)	0.79 (0.66–0.93)	0.59 (0.49–0.70)	0.58 (0.49–0.70)	0.59 (0.49–0.71)
Smoking during pregnancy						
No	1.00	1.00	1.00	1.00	1.00	1.00
Yes	2.59 (2.24–3.00)	1.90 (1.63–2.23)	1.91 (1.63–2.24)	2.92 (2.53–3.36)	2.23 (1.91–2.60)	2.23 (1.91–2.60)
Evidence of alcohol abuse						
No	1.00	1.00	1.00	1.00	1.00	1.00
Yes	8.65 (4.47–16.72)	4.06 (2.05–8.05)	4.12 (2.08–8.17)	2.62 (1.04–6.64)	1.09 (0.42–2.81)	1.10 (0.43–2.83)

a Adjusted for variables listed in the table;

b Adjusted for parity and variables listed in the table. CI, confidence interval; OR, odds ratio; SGA_WL_, small for gestational age defined as both birth weight and length <10th percentile.

**Table V:** Results of multivariable regression analyses for risk factors of SGA_W_ births in Murmansk County, Russia during 2006–2011

	SGA_W_, males, n=2,089			SGA_W_, females, n=1,950		
		
Characteristic	Crude OR	Adjusted OR^a^ (95% CI)	Adjusted OR^b^ (95% CI)	Crude OR	Adjusted OR^a^ (95% CI)	Adjusted OR^b^ (95% CI)
Age (years)						
<18	1.68 (1.21–2.34)	0.87 (0.61–1.23)	0.78 (0.55–1.11)	1.96 (1.44–2.67)	1.01 (0.72–1.42)	0.94 (0.67–1.32)
18–34	1.00	1.00	1.00	1.00	1.00	1.00
35+	0.89 (0.75–1.06)	1.10 (0.93–1.31)	1.27 (1.06–1.53)	0.71 (0.59–0.86)	0.93 (0.76–1.12)	1.03 (0.84–1.25)
Education in smokers/non–smokers						
None or primary, smoker	3.53 (2.60–4.77)	2.58 (1.88–3.55)	2.70 (1.96–3.71)	4.52 (3.44–5.94)	3.36 (2.51–4.51)	3.45 (2.57–4.63)
None or primary, non-smoker	2.66 (1.96–3.62)	2.15 (1.55–2.98)	2.23 (1.61–3.09)	2.18 (1.58–3.01)	1.76 (1.25–2.48)	1.80 (1.28–2.54)
Secondary, smoker	3.25 (2.80–3.78)	2.54 (2.15–2.99)	2.64 (2.23–3.09)	3.35 (2.87–3.91)	2.81 (2.37–3.32)	2.87 (2.42–3.39)
Secondary, non-smoker	1.58 (1.38–1.80)	1.34 (1.16–1.54)	1.36 (1.18–1.56)	1.44 (1.26–1.66)	1.28 (1.10–1.48)	1.29 (1.11–1.49)
Vocational school, smoker	2.16 (1.78–2.61)	1.85 (1.52–2.25)	1.88 (1.55–2.28)	2.15 (1.77–2.61)	1.96 (1.61–2.40)	1.99 (1.63–2.43)
Vocational school, non-smoker	1.26 (1.10–1.44)	1.19 (1.04–1.36)	1.21 (1.06–1.39)	1.25 (1.09–1.43)	1.23 (1.07–1.41)	1.24 (1.08–1.43)
Higher, smoker	1.38 (1.00–1.89)	1.33 (0.97–1.84)	1.32 (0.96–1.81)	1.34 (0.97–1.86)	1.33 (0.96–1.85)	1.32 (0.95–1.83)
Higher, non-smoker	1.00	1.00	1.00	1.00	1.00	1.00
Occupation						
Unemployed	1.65 (1.51–1.81)	1.30 (1.18–1.44)	1.28 (1.16–1.42)	1.56 (1.42–1.72)	1.19 (1.07–1.32)	1.18 (1.06–1.31)
Employed/ students	1.00	1.00	1.00	1.00	1.00	1.00
Marital status						
Unmarried	1.77 (1.61–1.94)	1.43 (1.29–1.58)	1.38 (1.24–1.52)	1.74 (1.58–1.92)	1.37 (1.23–1.52)	1.33 (1.19–1.47)
Married	1.00	1.00	1.00	1.00	1.00	1.00
Nutritional status						
Underweight	1.79 (1.54–2.09)	1.71 (1.46–2.00)	1.66 (1.42–1.94)	1.73 (1.48–2.03)	1.63 (1.39–1.91)	1.60 (1.36–1.87)
Normal weight	1.00	1.00	1.00	1.00	1.00	1.00
Overweight and obese	0.75 (0.67–0.84)	0.75 (0.67–0.84)	0.78 (0.70–0.88)	0.56 (0.49–0.63)	0.56 (0.49–0.63)	0.58 (0.51–0.65)
Evidence of alcohol abuse						
No	1.00	1.00	1.00	1.00	1.00	1.00
Yes	5.89 (3.26–10.62)	3.19 (1.74–5.85)	3.28 (1.79–6.02)	3.55 (1.87–6.71)	1.78 (0.92–3.44)	1.85 (0.96–3.58)

a Adjusted for variables listed in the table;

b Adjusted for parity and variables listed in the table. CI, confidence interval; OR, odds ratio; SGA_W,_ small for gestational age defined as birth weight <10th percentile.

After adjustment for studied variables, the risk of SGA_WL_ births of both sexes among low educated, unmarried, smoking or underweight women was higher compared with the corresponding reference groups (Table IV). Contrary to SGA_WL_ females, SGA_WL_ males were at 2.6-fold increased risk to be born to older (≥35 years) unemployed women. Evidence of alcohol abuse contributed to increased risk of SGA_WL_ males. Unemployed, unmarried and underweight mothers were at higher risk to deliver SGA_W_ boys and girls (Table V). Maternal overweight and obesity significantly related with lower risk of both SGA_WL_ and SGA_W_ births. In both sexes, risk of SGA_W_ birth was highest among smoking women with lower (none/primary and secondary) education (Table V).

After adjustment for parity maternal low education, unmarried status, smoking, as well as underweight continued to be associated with increased risk of SGA_WL_ births. Unemployed women aged 18–34 years and older than 35 years exhibited a 1.4- and 2.8-fold higher risk of giving birth to a boy being SGA_WL_, respectively. Mothers with evidence of alcohol abuse were at 4-fold higher risk to deliver SGA_WL_ boys (Table IV). Unemployment and unmarried status was associated with increased risk of SGA_W_ births. Smoking women with lower education were at high risk to deliver SGA_W_ infants. These results were similar for boys and girls (Table V). Overweight and obese women continued to demonstrate lower risk of both SGA_WL_ and SGA_W_ births compared with normal weight mothers. Risk of SGA_W_ boys’ birth was increased among women with evidence of alcohol abuse.

To assess if our results would be affected by also including stillbirths and infants with congenital birth defects, we additionally studied a cohort of 45,508 births. We obtained this cohort from the initial study population after application of all other exclusions shown in Fig. 1 except of stillbirths and birth defects. The proportions of term SGA_W_ and SGA_WL_ births were 9.5 and 4.2%, respectively, which did not differ from our findings presented above for a cohort where stillbirths and infants with birth defects were excluded. The 10th percentiles for BW and BL for both male and female infants remained unchanged except of the values of P10 for BW and BL in girls at GA of 37 weeks. BW and BL P10 values were 2,500 g and 47 cm in the cohort with stillbirths and infants with birth defects included, whereas the corresponding values in the cohort without abovementioned exclusions were 2,529 g and 48 cm. The results of regression analysis for both SGA_WL_ and SGA_W_ remained the same. Furthermore, the effect of studied risk factors did not change after inclusion of stillborn babies and infants with birth defects into the model.

## Discussion

In our study, the proportions of term SGA_W_ and SGA_WL_ were 9.7 and 4.1%, respectively. There is no implemented national birth register in Russia. Therefore, we used our study population as reference population to identify SGA births. Our results on the prevalence of SGA birth agree with the results based on data from the Kola Birth Registry, which demonstrated a 9.2% SGA prevalence (28). In contrast to the Kola Birth Registry, we found larger BW P10 values for both female and male infants for GA of 37–41 weeks. An explanation for the heavier babies in our study could be that we applied more exclusion criteria. We also predominantly used US estimation of GA in contrast to a combination of LMP and US data used in the Kola Birth Registry. An average difference of 2–3 days between LMP and US estimation was reported (35) with overestimation of GA based on LMP data (36).

The prevalence of SGA_W_ birth in our study was higher compared with the prevalence of 7.2% in a Dutch population (37). An even higher proportion of SGA_W_ (10.7%) was found in a multicentre cohort study (38), but that population encompassed both term and preterm infants. An 11.3% SGA_W_ prevalence was demonstrated in the same multicentre study when the additional years of observation were added (39). In a multi-ethnic New Zealand population, 11.8% of all births were SGA_W_ (13), and in Brazil, 13.1% of live born infants of both sexes were classified as SGA_W_ in a cross-sectional study (40). All the abovementioned studies used the ICD-10 criteria (1) to identify SGA birth.

The prevalence of SGA birth is expected to be lower compared with our and abovementioned studies if the internationally recommended definition of SGA infants (3) is applied. A study of the Swedish Medical Birth Register demonstrates 3.6% of infants with BW of more than 2 SDs below the mean for their GA (25). Data from Finland are in line with these results; 3.8% of all term and preterm newborns are classified as SGA (23). In our study, we applied the ICD-10 definition of SGA birth as it corresponds to national reports in Russia and, consequently, allowed us to compare our results with Russian national data. More strict criteria for SGA birth are implemented by the International SGA Advisory Board (3) and predominantly aim to identify children born SGA for growth hormone treatment. If we had applied criteria of BW of at least 2 SDs below the sex- and gestational age-specific mean, a total of 1,061 infants (2.4% in our cohort of 44,235 births) would be classified as SGA. As data on BW in MCBR were presented rounding up to dozens, the proportion of SGA births in our population was somewhat higher than 2.3% (equivalent to 2 SDs). Similar results are found based on birth register data of 533,666 singletons born between 1996 and 2008 in Finland (41). When internationally recommended criteria were used, 2.6% of term boys were classified as SGA_W_. The largest proportion (3.8%) was reported for singleton boys born at 37 weeks (41).

Our results showed that unfavourable social factors increased the risk of SGA. These results are consistent with the findings of Ota et al. (4) who demonstrate an association between socio-demographic status and term SGA. Our findings regarding higher proportion of young mothers in the SGA group correspond to a study from Brazil, which found the largest proportion (15.6%) of women aged <20 years among those who delivered SGA infants (40). Data from a multicountry survey on maternal and newborn health demonstrate a prevalence of young mothers of 17.8% in term SGA births (4). The same study also shows that 28.8% of all mothers in the preterm SGA group were <20 years old. In a study from New Zeeland, 14% of mothers who delivered SGA babies were younger than 20 years. In contrast, the proportion of mothers aged 20–29 years in the SGA group was 11.8% (13).

Maternal age is suggested as a possible explanatory factor for SGA in mothers with different educational levels, with mean maternal age being lowest in low-educated women. The proportion of SGA births among these mothers is higher compared with high-educated women (37). On the contrary, McCowan et al. (38) and Khashan et al. (39) found no evidence of difference in mothers’ mean age between SGA and non-SGA groups. Their findings correspond to recently published Finnish results (14), which report even less risk of SGA among mothers ≤19 years old compared with older women. In our study, we found that the effect of maternal age continued to be statistically significant after multivariable adjustment only for SGA males. In contrast to a previously published study that does not confirm sex difference as SGA risk factor (40), we showed different contribution of studied factors to birth of SGA males and females.

Our findings of higher proportion of low-educated mothers in both SGA_WL_ and SGA_W_ groups compared with non-SGA infants are consistent with a study from the Netherlands (37) which shows an almost two-fold higher prevalence of SGA_W_ in low-educated women compared with high-educated women. Other studies also confirm higher proportion of low-educated mothers in the SGA group compared with non-SGA births (4, 27, 40). In a study from Canada that investigated the effect of maternal education on different perinatal outcomes including SGA birth, the adjusted ORs demonstrated apparent risk gradients across the maternal education strata for SGA birth. Higher rates of SGA birth were found among women with lower educational levels (27). In this study, we demonstrated that the effect of low education on SGA birth remained significant after adjustment for other factors. Smoking, low-educated women were at highest risk to deliver SGA_W_ boys and girls. Van den Berg et al. (37) highlighted that the association between maternal education and SGA was not independent; maternal smoking overruled the contribution of other factors including maternal education. Therefore, implementation of more effective programmes aimed on smoking cessation among low-educated women may result in reduction of SGA birth (37).

Results regarding marital status and SGA birth are conflicting. Whereas we found that single mothers were at higher risk of SGA birth, others have also reported that unmarried maternal status or living without spouse was associated with an increased risk of SGA (4, 14, 42). Rates of single mothers did neither differ between SGA and non-SGA groups in an international prospective study (38) nor in a study from Canada (43). However, Canadian-born unmarried mothers are, compared with married mothers, at higher risk of delivering SGA infants irrespective of the interpregnancy interval duration (43).

While some studies show that maternal residence in urban areas or large cities increase the risk for SGA births (25, 44), other studies (42) including the current one found no such association in multivariable logistic regression analysis. In fact, using chi-squared testing we demonstrated higher proportion of SGA_WL_ births in women living in rural areas compared with those living in cities. One reason for this difference could be a limited availability of and access to medical facilities for rural citizens of Murmansk County, before the implementation of three-level system of perinatal care in 2008. Beginning that year, women with high-risk pregnancies were required to receive care at a Level III delivery hospital that was properly equipped for managing complicated pregnancies and deliveries. Level I hospitals are located mostly in rural areas and are reserved for low-risk pregnant women and newborns. Misclassification of urban areas as rural territories might also be a confounder in our study. Sixteen cities in Murmansk County were presented on the official website of Murmansk County (www.gov-murman.ru) in 2011, which was the year the data collection in MCBR ended. Smaller settlements with high-developed medical service were not included in the list. Therefore, these settlements might be misclassified as rural areas in this study.

Consistent with findings from a previous study (22), unemployed mothers in our study were at higher risk of SGA birth in comparison with those employed. Not only unemployment of an individual but unemployment at municipality level contributes to SGA birth (23). In contrast, a large European cohort study (26) demonstrates that maternal overall employment during pregnancy contributes to a higher risk of SGA.

Our findings that smoking and evidence of alcohol abuse significantly associated with SGA birth are in line with other studies (13, 14, 15, 19, 25, 37). However, in contrast to results by Van den Berg et al. (37), inclusion of smoking and evidence of alcohol abuse into our model did not reduce or nullify the effect of other social factors. In our study, multivariable adjustment slightly decreased the odds for smokers to deliver SGA_WL_ infants, but it still remained high; adjusted ORs in SGA_WL_ males and females groups were 1.91 and 2.23, respectively. These ORs correspond to results of others (40). In our study, the proportion of smoking mothers was two-fold higher in both SGA groups compared with non-SGA infants. Li et al. (25) report 1.4-times increased risk in smoking mothers. We demonstrated increased risk of SGA_W_ births among smokers at any level of maternal education. A study from the Netherlands (37) demonstrates even higher risk in smokers (OR=3.06) but does not confirm the effect of alcohol on risk of SGA birth. We detected 7- and 5-fold difference in the proportions of alcohol abusing mothers in SGA_WL_ and SGA_W_ groups, respectively, compared with non-SGA births. The odds to have term SGA_WL_ and SGA_W_ boys is, respectively, 4- and 3-times higher among alcohol abusing mothers compared with those not being alcohol dependent. In contrast to our findings, the results of a meta-analysis show non-significant effect of alcohol in studies adjusted for confounders (19). The same study also reports a dose–response relationship between mother's alcohol consumption and SGA birth. In our study, we could not assess the effect of dose, as data on amount of alcohol intake were not recorded in the MCBR.

In this study, higher risk of both SGA_W_ and SGA_WL_ was associated with maternal underweight. These findings are in line with earlier studies from other countries (14, 15, 16, 37). A study from Finland (14) reports 1.4-times higher risk of SGA in mothers having pre-pregnancy BMI≤24.9 kg/m^2^ compared with overweight women. We found that overweight and obesity decreased the risk of SGA births irrespective of SGA definition. In fact, high maternal BMI does not play a role as a protective factor; obese women have elevated risk of foetal macrosomia, which is caused by both an increase in the foetus size and changes in its body composition (45, 46). Decreased foetal lean body mass and increased fat mass may lead to adverse health outcomes in offspring of overweight/obese mothers (47). In fact for foetal macrosomia, maternal obesity is suggested as main factor followed by pre-gestational diabetes (48). A study from New Zeeland (13) demonstrates that obesity associates with increased risk of SGA birth as identified by customised BW centiles in contrast to SGA defined on the basis of population BW references. Weight gain during pregnancy may also associate with both increased infant fat mass and body fat as pre-pregnancy BMI accounts for approximately 7% of the observed variations of these two parameters (47).

### Strengths and limitations

The main strength of our study is the use of a birth registry including socio-demographic information, detailed data about maternal medical history and infant's health. The initial MCBR database included the entire population of mothers and newborns in Murmansk County over a period of 6 years. Individual data on maternal smoking and alcohol consumption considered as strong predictors of SGA birth (13, 37) were available in the MCBR.

To investigate possible differences in associations between a set of selected social factors and SGA_WL_ and SGA_W_, we applied two definitions of SGA that are widely used in practice. The possibility to investigate multiple outcomes is one of the strengths of any cohort study (49).

In our study, we did not examine the contribution of medical conditions or other predictors to SGA births, which other studies have focused on (4, 13, 42). In addition, we could not investigate pre-pregnancy BMI due to limitations of available data. Early pregnancy BMI was considered applicable in our study, as a previously published study shows that both mean maternal weight and body composition do not change during early pregnancy (50).

We used sex-specific P10 values for both BW and BL as this may improve identification of SGA infants (51). Data on smoking and alcohol consumption in the MCBR are partly self-reported, based on mothers’ reports during pregnancy and medical staff records. Women's unwillingness to disclose information may lead to underreporting. Underreporting and missing information are commonly observed in retrospective cohort study (49).

## Conclusions

We found social disparities in SGA birth at the individual level in a Russian Arctic setting. Maternal low education, unemployment, unmarried status and underweight carried a significantly higher risk of term SGA births irrespective of SGA definition. Smoking and evidence of alcohol abuse are also associated with SGA birth. Therefore, early identification of women with the above risk factors and implementation of public health programmes aimed at reducing smoking, alcohol consumption and underweight before and in early pregnancy may potentially result in reduction of SGA births.
